# Heat Exhaustion and Heat Stroke Among Active Component Members of the U.S. Armed Forces, 2019-2023

**Published:** 2024-04-20

**Authors:** Alexis L. Maule, Kiara D. Scatliffe-Carrion, Katherine S. Kotas, Jacob D. Smith, John F. Ambrose

**Affiliations:** 1 Disease Epidemiology Program, Defense Centers for Public Health–Aberdeen, Defense Health Agency, Aberdeen Proving Ground, MD

## Abstract

The most serious types of heat illnesses, heat exhaustion and heat stroke, are occupational hazards associated with many of the military’s training and operational environments. These illnesses can typically be prevented by appropriate situational awareness, risk management strategies, along with effective countermeasures. In 2023, the crude incidence of heat stroke and heat exhaustion were 31.7 and 172.7 cases per 100,000 person-years, respectively. The rates of incident heat stroke declined during the 2019 to 2023 surveillance period, but rates of incident heat exhaustion increased over the same period. In 2023, higher rates of heat stroke were observed among male service members compared to their female counterparts, and female service members experienced higher rates of heat exhaustion compared to male personnel. Heat illness rates were also higher among those younger than age 20, Marine Corps and Army service members, non-Hispanic Black service members, and recruits. Leaders, training cadres, and supporting medical and safety personnel must inform their subordinate and supported service members of heat illness risks, preventive measures, early signs and symptoms of illness, and appropriate interventions.

**What are the new findings?:**

From 2019 to 2023, the crude annual incidence rate of heat stroke decreased 15.6%, while the crude annual incidence rate of heat exhaustion increased by 4.6% during the same period. The observed decrease in heat stroke coincident with observed increase in heat exhaustion may demonstrate evidence of successful awareness and prevention efforts emphasizing recognition of the early signs and symptoms of less severe heat illness, which resulted in avoidance of more severe illness such as heat stroke. This is the first heat illness surveillance report that includes U.S. Coast Guard and Space Force data. Consequently, a comparison of the crude annual rates for 2019-2023 in this report are not directly comparable to those published in previous reports.

**What is the impact on readiness and force health protection?:**

Many factors inherent to military training and operational environments can increase risk of heat illness: environmental factors (e.g., heat and humidity), occupational factors (e.g., strenuous activity and intense training), and personal factors (e.g., highly motivated individuals and populations). Despite these risks, heat illness incidence and severity can be reduced by implementing and enforcing appropriate countermeasures. Units that fail to implement heat illness mitigation measures risk impeding or interrupting training programs, resulting in otherwise preventable reductions in operational tempo or critical mission failure due to lost personnel and resources.

## 1 BACKGROUND

Heat illnesses, which can drastically affect both individual and unit readiness, continue to contribute significantly to annual morbidity within the active component of the U.S. Armed Forces. Heat illness refers to a group of disorders that result from a disruption of thermoregulation due to heat stress caused by environmental heat exposure, high energy expenditure (i.e., metabolic heat production), or a combination of both factors.^1,2,3,4^ Metabolic heat production increases during prolonged engagement in strenuous physical activity, and additional exposure to environmental heat stress elevates core and skin temperatures.^2,3^ Documented measures to reduce the risk of heat illnesses include, but are not limited to, electrolyte intake, adherence to fluid replacement and work-rest guidelines, and body cooling methods (e.g., arm immersion cooling systems).^3,5^ Identifying high-risk service members, such as those who are unacclimatized, taking certain medications (e.g., antihistamines, tricyclic antidepressants, ADHD medication, beta-blocker antihypertensives), have low physical fitness, or were previously diagnosed with heat illness, is also critical in reducing morbidity due to heat illnesses.^3^

Heat illness occurs within a continuum of severity, from less severe (e.g., heat cramps, rash, edema, and syncope), then to heat exhaustion, followed by potentially life-threatening (e.g., heat stroke). Heat exhaustion and heat stroke are reportable medical events (RMEs) in the U.S. Military Health System (MHS). All occurrences that require medical intervention or result in change of duty status must be reported.^6^

Specific signs and symptoms that characterize heat illnesses allow initial recognition of their occurrence in the field and subsequent identification of a heat illness that should be reported. A confirmed case of heat exhaustion must fulfill 3 conditions, during or immediately following exertion or heat exposure: 1) evidence of elevated core body temperature (not greater than 104°F/40°C), 2) short-term physical collapse or debilitation, with 3) no significant central nervous system dysfunction.^7^ Acute dehydration often accompanies heat exhaustion but is not required for diagnosis.^8^ If any central nervous system dysfunction develops (e.g., dizziness or headache), it should be mild and rapidly resolve with rest and cooling measures, otherwise the patient may be experiencing a heat stroke.^7,8^

Heat stroke is a debilitating and potentially life-threatening condition characterized by severe hyperthermia. During a period of heat exposure or exertion, a probable case of heat stroke requires 1) evidence of elevated core body temperature and 2) central nervous system dysfunction (e.g., change in mental status, delirium, stupor, loss of consciousness, or coma). A confirmed case of heat stroke requires verification and documentation of a core body temperature of 104ºF/40ºC or greater with central nervous system dysfunction.^8,9^ The onset of heat stroke should prompt aggressive intervention featuring rapid cooling (e.g., iced sheets) and supportive therapy such as fluid replacement only when previous fluid intake can be confirmed, to avoid water intoxication (i.e., hyponatremia).^8,9,10^ Multiple organ failure is the ultimate cause of mortality from heat stroke.^9^

Ongoing surveillance of heat illnesses is necessary to determine if prevention guidelines and countermeasures are working, in addition to identifying high-risk groups and activities that may lead to heat illness. Since 2001 the *MSMR* has published regular updates on the incidence of heat illness among U.S. active component service members (ACSMs). This update presents summaries of heat stroke and heat exhaustion case counts, incidence rates, and locations between 2019 and 2023.

## 2 METHODS

The surveillance population for this analysis includes all individuals who served in the active component of the Army, Navy, Marine Corps, Air Force, Space Force, or Coast Guard at any time during the surveillance period of January 1, 2019 through December 31, 2023. Space Force data are only complete for 2023.

All data used to determine incident heat illness diagnoses were derived from 4 sources: MHS Management, Analysis and Reporting Tool (M2), Defense Medical Surveillance System (DMSS), Disease Reporting System internet (DRSi), and Theater Medical Data Store (TMDS). Heat illness cases were identified using specific diagnostic codes from the ambulatory care encounters and hospitalizations of ACSMs in fixed military and civilian (if reimbursed through the MHS) hospitals and clinics worldwide. In addition to medical encounter data, heat illness medical event reports were identified in DRSi, including information on hospitalization status (yes/no). If a heat illness was reported in DRSi, but not found in the medical record, the case was still counted. For example, an individual could be treated in the field by a medic for mild or non-life-threatening heat illness without a recorded medical encounter, but the case is deemed a reportable heat exhaustion because of symptoms observed in the field.

In this update, a case of heat illness was defined as an individual with 1) a hospitalization or outpatient medical encounter record with a primary (first-listed) or secondary (second-listed) diagnosis of heat stroke (International Classification of Diseases, 9th Revision [ICD-9]: 992.0; ICD, 10th Revision: T67.0*) or heat exhaustion (ICD-9: 992.3–992.5; ICD-10: T67.3*–T67.5*) or 2) a RME record of heat exhaustion or heat stroke.^11^ The asterisk denotes that all subsequent digits or characters noted in the diagnostic code were included in the identification of ICD-10 codes (e.g., T67.3XXA).

An individual was considered a case of heat illness only once per calendar year. If a service member had diagnoses for both heat stroke and heat exhaustion during a given year, the more severe diagnosis (heat stroke) was selected. If a service member had inpatient and outpatient encounters for heat stroke or heat exhaustion, the inpatient encounter was prioritized over the outpatient visit, when identifying hospitalized cases. Within a calendar year, if an individual had a diagnostic code that denoted a subsequent encounter (i.e., ICD-10 7th digit equal to “D”) or an encounter for sequelae (i.e., ICD-10 7th digit equal to “S”), but had no diagnostic codes indicating an initial visit (i.e., ICD-10 7th digit equal to “A”), the case was removed to avoid over-estimating heat illness cases by including those receiving follow-up care.

For health surveillance purposes, recruits were identified as ACSMs assigned to service-specific training locations and basic training periods using an algorithm based on age, rank, and time in service. Recruits were considered a separate category of enlisted service members in summaries of heat illnesses by military grade. In summaries of heat illness by location, the Defense Medical Information System Identifier (DMIS ID) was utilized to determine the installation or geographic location of diagnosis and medical treatment. Heat illness cases from the U.S. Central Command (CENTCOM) area of responsibility (AOR) were reported separately and identified with specific combatant command or country codes.

In-theater diagnoses of heat illness were identified from medical records of deployed service members whose health care encounters were documented in TMDS, and the same case-defining criteria and incidence rules described previously were applied to those encounters. Evacuations were identified if the service member had a follow-up inpatient encounter in a permanent military hospital or clinic in the U.S. or Europe, from 5 days preceding until 10 days following their heat illness event.

Incidence rates were calculated as incident cases of heat illness per 100,000 ACSM person-years (p-years). Percent change in incidence was calculated using unrounded rates. Because reporting heat exhaustion and heat stroke cases is required, the proportion of outpatient and inpatient cases that also had a report in DRSi was calculated.^4^

## 3 RESULTS

In 2023, the MHS reported 415 cases of heat stroke, resulting in a crude incidence rate of 31.7 cases per 100,000 p-yrs (**Table 1**). Subgroup-specific incidence rates of heat stroke were highest among men, those younger than age 20, non-Hispanic Black service members, Marine Corps and Army personnel, recruits, and those in combat-specific occupations.

**Figure 1:**
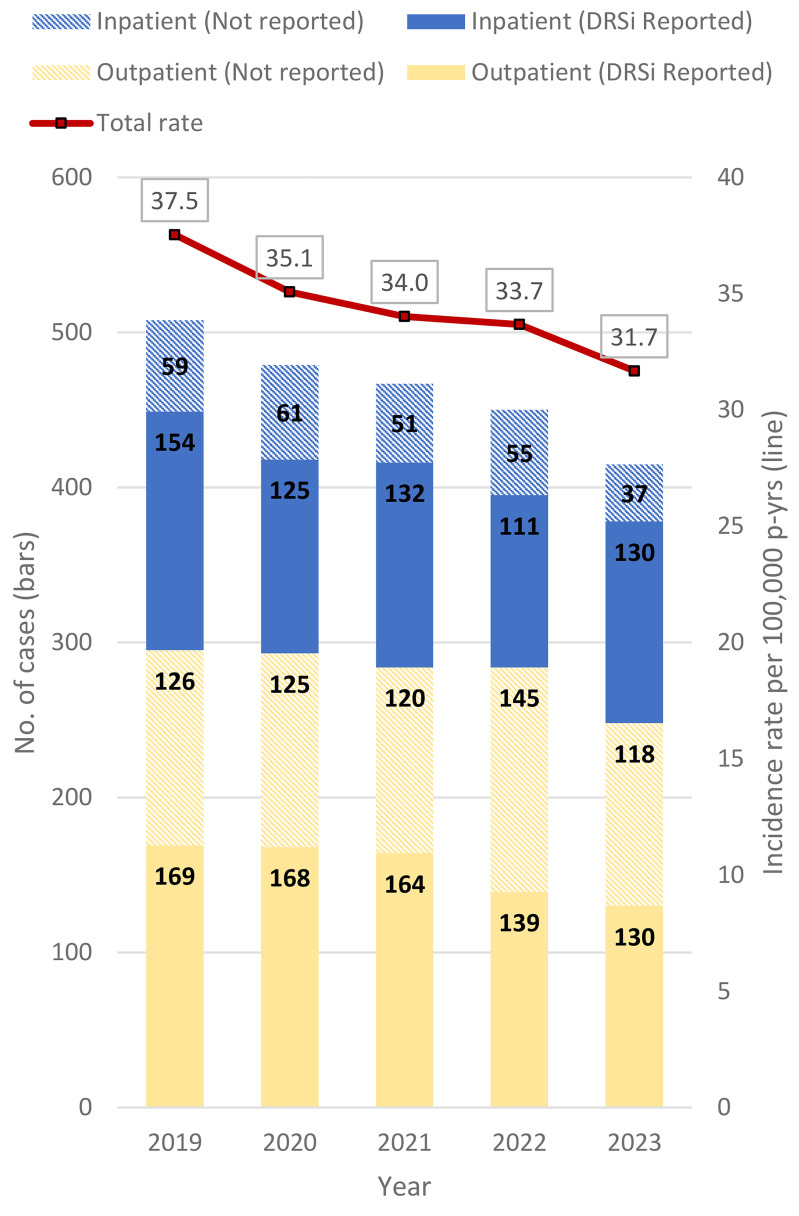
Incident Cases^a^ and Incidence Rates of Heat Stroke, by Encounter Type and Year of Diagnosis, Active Component, U.S. Armed Forces, 2019–2023

The crude annual incidence rate of heat stroke decreased 15.6% (**Figure 1**) from 2019 through 2023, with a 5.9% decline from 2022 to 2023. Meanwhile, the proportion of heat stroke cases that were hospitalized increased from 36.9% in 2022 to 40.2% in 2023. Of all inpatient heat stroke cases, 71.3% were reported to DRSi, while just over half (54.8%) of outpatient heat stroke cases had a medical event report in DRSi.

The 2,263 cases of heat exhaustion in 2023 correspond to a crude incidence rate of 172.7 cases per 100,000 p-yrs (**Table 1**). Unlike heat stroke, where higher rates were observed among male service members, the rate of heat exhaustion was 24% higher among female service members. Similar to heat stroke, with the exception of the difference in rates by sex, higher rates of heat exhaustion were noted for personnel younger than age 20, non-Hispanic Black service members, Marine Corps and Army personnel, and recruits. The incidence rate of heat exhaustion among recruits was 12.5 and 13.5 times higher than other enlisted service members and officers, respectively.

**Figure 2:**
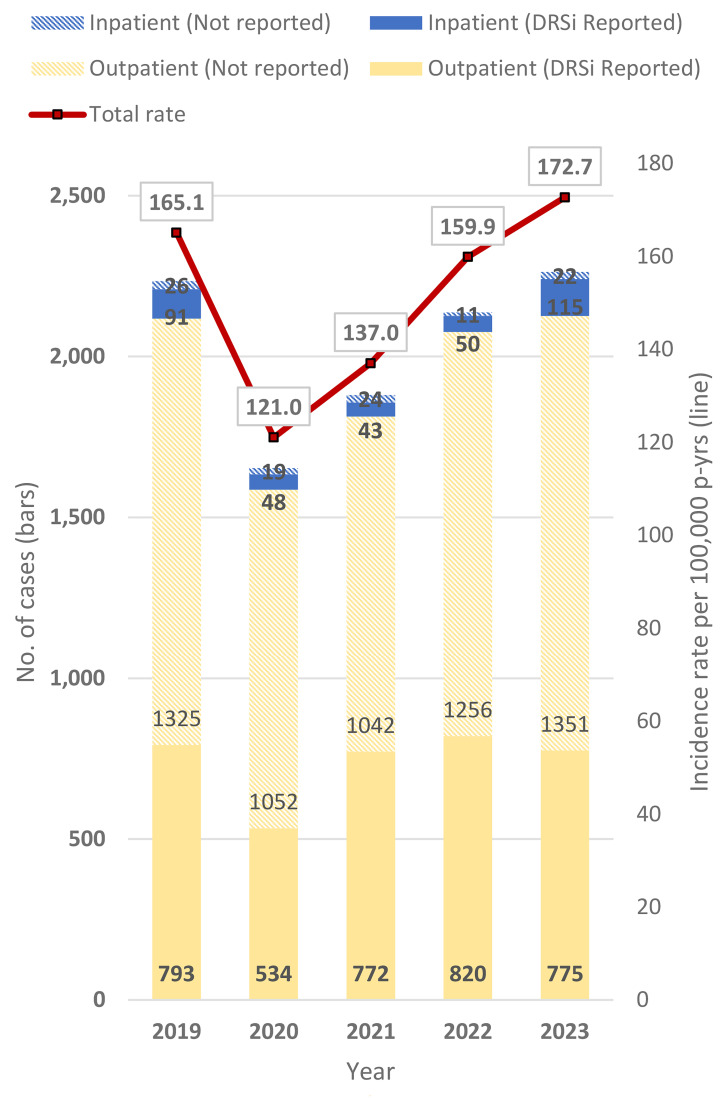
Incident Cases^a^ and Incidence Rates of Heat Exhaustion, by Encounter Type and Year of Diagnosis, Active Component, U.S. Armed Forces, 2019–2023

Between 2019 and 2023, the crude annual incidence rate of heat exhaustion increased 4.6%, including an 8.0% increase from 2022 to 2023 (**Figure 2**). The proportion of heat exhaustion cases that were hospitalized also increased from 2022 (2.8%) to 2023 (6.0%). Over three-quarters of the inpatient heat exhaustion cases (77.3%) were reported in DRSi, while only 38.0% of outpatient heat exhaustion cases had a medical event report in DRSi.

**Heat illnesses by location**

During the 5-year surveillance period, 12,488 heat illnesses were diagnosed at more than 250 military installations and geographic locations worldwide (**Table 2**). Of these heat illness cases, 5.8% occurred outside the U.S., including 282 in Okinawa, Japan. Between 2019 and 2023, 21 locations reported at least 100 cases of heat illness, and those locations accounted for over three-quarters (77.5%) of all active component cases. Three Army installations: Fort Moore, GA; Fort Liberty, NC; Ft Campbell, KY, and 2 Marine Corps bases, MCB Camp Lejeune/Cherry Point, NC, and MCRD Parris Island/Beaufort, SC, accounted for 40.4% of the total heat illnesses during the surveillance period. Of the 21 locations with at least 100 cases of heat illness, 13 are in the southern U.S.

**Heat illnesses in CENTCOM AOR**

**Figure 3:**
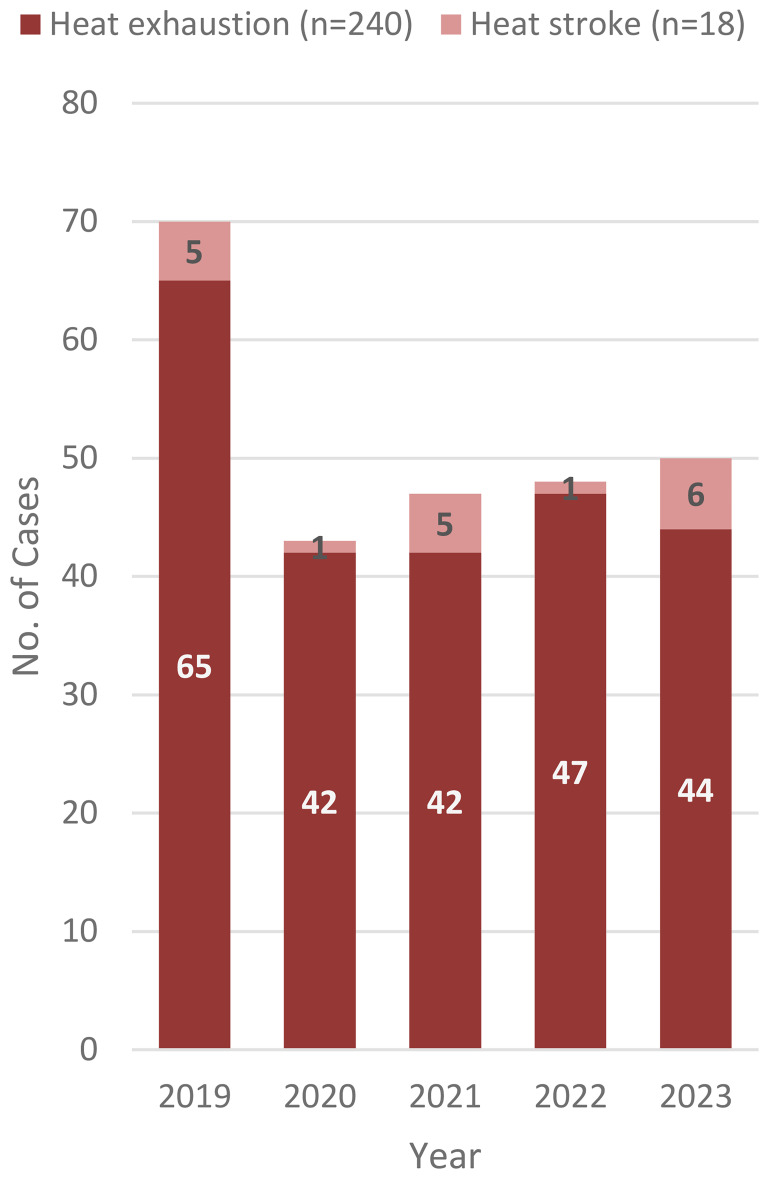
Incident Cases of Heat Illnesses in the CENTCOM AOR, Active Component, U.S. Armed Forces, 2019–2023

During the 5-year surveillance period, there were 258 cases of heat illness in the CENTCOM AOR (**Figure 3**). Of the cases of heat illness, 7.0% (n=18) were heat stroke. Cases of heat illness occurred most frequently among deployed service members who were male (n=194, 75.2%), 20-24 years old (n=125, 48.4%) and in the Army (n=86, 33.3%) or Navy (n=85, 32.9%) (data not shown). During the surveillance period, 3 service members were medically evacuated for heat illnesses from the CENTCOM AOR: 1 evacuation occurred in November 2020, 1 in August 2022, and 1 in July 2023 (data not shown).

## 4 DISCUSSION

Over the 5-year surveillance period, the rate of heat stroke decreased annually from 2019 to 2023, while the rate of heat exhaustion dropped in 2020 compared to 2019 and then increased annually from 2021 to 2023. Between 2022 and 2023, the increase in the heat exhaustion rate (8.0% rate increase) was greater than the decrease in the heat stroke rate (5.9% rate decrease). While the reason for the observed decrease in heat stroke and observed increase in heat exhaustion is unknown, current heat illness prevention guidelines emphasize education about the signs, symptoms, and management of heat casualties, with the goal of preventing more severe heat illness (i.e., heat stroke).^3,5,12^ Earlier recognition of what constitutes a heat illness may increase the likelihood of identifying a case of heat exhaustion.^13^

While the required reporting of inpatient cases for both heat stroke and heat exhaustion is more complete than outpatient case reporting, which is also required, the proportion of all cases reported to DRSi remained about 40%, on average, throughout the 5-year reporting period. It is possible that treatment providers are unaware of the reporting criteria, which provides opportunities to improve providers’ awareness of DRSi as the system of record for DOD RMEs and the specific requirements for reporting heat illnesses. Additionally, the diagnosis and diagnostic coding of similar heat-related clinical illnesses can be subjective and subject to errors, and clinical definitions for heat stroke and heat illness differ somewhat from surveillance definitions.^14^

This report includes data from the Coast Guard and Space Force active components for the first time. While the personnel in these services represent a combined average of about 3-4% of the ACSM population in the U.S. Armed Forces, they account for less than 1% of heat illness cases. The inclusion of Coast Guard and Space Force personnel may have a slight dampening effect on heat illness rates when reviewing U.S. Armed Forces rates in prior *MSMR* reports; comparing heat illness rates presented in this report to those in previous *MSMR* reports is not advised.

There are limitations to this update that should be considered when interpreting its findings. Although heat illnesses were summarized by the location of diagnosis or report, medical care may not occur at the same location (i.e., installation) as the heat illness event, particularly if the case required a level of care not available locally. To account for locations with significant medical care redundancy, some installations were combined (e.g., MCB Camp Lejeune/Cherry Point, NC, in **Table 2**); this merging of locations was most prevalent with Marine Corps and Navy locations.

The method used to identify recruits likely resulted in some misclassification of recruit training status. The algorithm did not account for the additional training time in Army’s One Station Unit Training beyond the traditional basic combat training period. In addition, there was likely incomplete capture of heat illnesses treated in the field during training and deployments, rather than at a fixed military hospital or clinic; this may be particularly true for heat exhaustion cases when symptoms rapidly resolve after a period of rest. Finally, military hospitals and clinics transitioned to MHS GENESIS in waves from February 2017 to March 2024, and the reliability and completeness of MHS GENESIS data are still being evaluated. Gaps in diagnosis codes have been identified, which may result in clinical event under-reporting through medical encounter data.

Maintaining regular heat illness surveillance helps identify the magnitude of the impact these conditions have on service member health, training, and force readiness. At the command and unit level, emphasis on evidence-based prevention, mitigation and risk management, with continued education on the signs, symptoms, and early field interventions for heat illness, are crucial steps in reducing the impact of heat illness morbidity on the force. To ensure protection throughout the force, DOD standards, policies, or procedures should determine the prevention, mitigation, and management of heat illnesses.

## Figures and Tables

**Table 1:** Incident Cases^a^ and Incidence Rates^b^ of Heat Illness, Active Component, U.S. Armed Forces, 2023

	**Heat Stroke**		**Heat Exhaustion**		**Total Heat Illness Diagnoses**	
	No.	Rate^b^	No.	Rate^b^	No.	Rate^b^
**Total**	415	31.7	2,263	172.7	2,678	204.4
** Sex**						
Male	361	33.4	1,790	165.8	2,151	199.2
Female	54	23.4	473	205.2	527	228.7
** Age group**, y						
<20	59	72.2	623	762.7	682	834.9
20-24	191	47.7	936	233.7	1,127	281.4
25-29	101	32.9	399	130.0	500	162.9
30-34	40	18.4	179	82.3	219	100.6
35-39	19	11.3	83	49.4	102	60.8
40+	5	3.7	43	31.8	48	35.5
** Race and ethnicity**						
White, non-Hispanic	224	32.4	165.3	34.50	1,367	197.7
Black, non-Hispanic or African American	74	35.6	394	189.4	468	224.9
Hispanic or Latino	63	25.2	459	183.3	522	208.4
Other/unknown^c^	54	33.7	267	166.7	321	200.4
** Service**						
Army	243	54.2	1,232	274.6	1,475	328.8
Navy	34	10.3	173	52.7	207	63.0
Air Force	22	7.0	363	115.0	385	121.9
Marine Corps	113	66.5	481	283.2	594	349.7
Space Force	0	-	8	93.6	8	93.6
Coast Guard	3	7.7	6	15.5	9	23.2
** Military status**						
Recruit trainees	33	391.3	625	7,411.9	658	7,803.2
Enlisted	306	28.9	1,429	135.1	1,735	164.0
Officer	76	31.2	209	85.7	285	116.9
** Military occupation**						
Combat-specific^d^	152	91.1	476	285.3	628	376.4
Motor transport	12	27.5	41	93.9	53	121.4
Pilot/air crew	4	8.8	9	19.7	13	28.4
Repair/engineering	21	5.7	124	33.4	145	39.1
Communications/intelligence	10	3.6	23	8.2	33	11.8
Health care	20	18.9	75	71.0	95	89.9
Other/unknown	196	65.7	1,515	507.6	1,711	573.3

Abbreviations: No., number; y, years.

^a^One case per person per calendar year.

^b^Rate per 100,000 person-years.

^c^Includes those of American Indian/Alaskan Native, Asian, Native Hawaiian/Pacific Islander, and unknown race and ethnicity.

^d^Infantry/artillery/combat engineering/armor.

**Table 2:** Heat Illness Events^a^ by Location of Diagnosis or Report (with minimum 100 cases during the period), Active Component, U.S. Armed Forces, 2019–2023

**Location of Diagnosis**	**No.**	**% Total**
Fort Moore, GA	2,072	16.6
MCB Camp Lejeune/Cherry Point, NC	941	7.5
Fort Liberty, NC	817	6.5
Fort Campbell, KY	630	5.0
MCRD Parris Island, SC	589	4.7
JB San Antonio, TX	580	4.6
Fort Johnson, LA	500	4.0
MCRD San Diego/NB San Diego, CA	491	3.9
Fort Cavazos, TX	456	3.7
MCB Camp Pendleton, CA	391	3.1
MCB Quantico, VA	322	2.6
Fort Jackson, SC	314	2.5
Fort Sill, OK	282	2.3
Okinawa, Japan	282	2.3
Twentynine Palms, CA	186	1.5
Fort Stewart, GA	163	1.3
Fort Irwin, CA	159	1.3
Fort Shafter, HI	157	1.3
Fort Leonard Wood, MO	145	1.2
Fort Riley, KS	104	0.8
Fort Bliss, TX	101	0.8
Outside U.S.^b^	446	3.6
All other locations	2,359	18.9
Total	12,488	100.0

Abbreviations: No., number; MCB, Marine Corps Base; MCRD, Marine Corps Recruit Depot; NB, Naval Base; NH, Naval Hospital; JB, Joint Base.

^a^One heat illness per person per year.

^b^Excluding Okinawa, Japan.

Note: Initial entry recruit training locations include Fort Jackson, Fort Leonard Wood, Fort Moore, Fort Sill, MCRD Parris Island, MCRD San Diego/NB San Diego, and JB San Antonio. Fort Johnson is the Joint Readiness Training Center (JRTC) and Fort Irwin is the National Training Center (NTC).

## AUTHOR AFFILIATIONS

Disease Epidemiology Program, Defense Centers for Public Health–Aberdeen, Defense Health Agency, Aberdeen Proving Ground, MD
